# Maternity Continuum Care Completion and Its Associated Factors in Northwest Ethiopia

**DOI:** 10.1155/2022/1309881

**Published:** 2022-02-17

**Authors:** Daniel Tsega, Melaku Admas, Asmare Talie, Tesfa Birlew Tsega, Molla Yigzaw Birhanu, Simegn Alemu, Belayneh Mengist

**Affiliations:** ^1^Department of Midwifery, Medicine and Health Science College, Wolkite University, Wolkite, PO Box 07, Ethiopia; ^2^Department of Midwifery, Medicine and Health Science College, Debre Markos University, Debre Markos, PO Box 269, Ethiopia; ^3^Department of Public Health, Medicine and Health Science College, Debre Markos University, Debre Markos, PO Box 269, Ethiopia

## Abstract

**Background:**

Continuum care is a basic package approach for women to receive essential services throughout pregnancy, childbirth, and postpartum, and it is critical for women and their infants' survival and well-being. Although it is an effective strategy for improving maternal and child health, it has not been implemented adequately in less developed countries, primarily in sub-Saharan Africa, including Ethiopia, where 55% of women have been dropped out from the continuum of care. Therefore, this study is aimed at assessing maternity continuum care completion and its associated factors within northwest Ethiopia, 2020.

**Materials and Methods:**

A community-based cross-sectional study design was considered among 504 women from March 10 to March 30, 2020, using pretested and structured questionnaires administered via face-to-face interviews. To select study participants, a simple random sampling technique was used. Data were coded, checked, and entered into EpiData software (V. 4.2), then transferred to SPSS (V. 25) for further analysis. A bivariable analysis with 95% CI was performed, and variables with *P* 0.25 during binary logistic regression were entered into a multivariable analysis to assess predictors' independent effect.

**Results:**

About 177 (37.6%) women completed maternal continuum care. Women with secondary education and above (AOR = 2.75, 95% CI 1.42-5.32), urban residence (AOR = 2.45, 95% CI 1.35-4.45), using ambulance transport (AOR = 3.96, 95% CI 2.19-7.19), mass media exposure (AOR = 3.64, 95% CI 2.02-6.56), and distance from health facilities (AOR = 3.22, 95% CI 1.84-5.63) showed significant positive associations with completion of maternity continuum care.

**Conclusion:**

However, a higher proportion of mothers completed the continuum of maternity care in the district than Ethiopian Demographic and Health Survey 2016 (9.1%); further interventions are compulsory to reach the acceptable level. Hence, comprehensive awareness-raising, education, and promotion activities at the community and health facility levels and empowering women in health care and decision-making backing to expand the completion of maternity continuum of care are necessary.

## 1. Introduction

Continuity of maternity care is a key program strategy that women have received as a continuation of care throughout the life cycle of pregnancy, childbirth, and postpartum periods and that promotes the improvement of women's status and their neonatal health in the field of global health [1–3]. Proper maternal and neonatal morbidity and mortality [4, 5] are the main interventions that women receive during the continuum of care and are crucial to the survival and well-being of both the mother and the child [6–8]. The idea of a continuum of care has two dimensions (time and place). Continuity of care in its time dimension refers to a situation in which a woman receives maternal health services throughout the life cycle of pregnancy, during adolescence, including family planning, education, nutrition, and empowerment of girls; pregnancy; childbirth; postpartum; childhood; and pregnancy. Alternatively, the emphasis is on combining the family, community, and institutional levels of maternal health services [2, 9].

According to World Health Organization (WHO) standards, at least four antenatal care (ANC) visits are recommended [10, 11], and recently, in 2016, WHO updated ANC visits; that is, 8 ANC contacts are advised to cut perinatal death and women's experience of care, but it is not still applied in Ethiopia [12]. The continuum of maternal and child care is an effective policy to improve maternal and child health; however, it has not been sufficiently implemented in low- and middle-income countries especially in sub-Saharan African countries including Ethiopia [13].

Ethiopia has established a number of strategies and programs to enhance and improve the use of facilities for health care services such as FP, ANC, institutional delivery, and PNC [14, 15]. Despite efforts to improve the use of maternity health services, the continuum of maternity health services remains shallow in Ethiopia, which needs more investigation [4]. Identifying the magnitude of the continuum of care and associated factors is therefore important for interventions to reduce failure in the continuum of care.

## 2. Materials and Methods

### 2.1. Study Design, Area, and Period

This community-based cross-sectional study was conducted between 10 and 30 March 2020 in the Ebinat district, one of the southern Gondar districts in the Amhara region of Ethiopia. It is located 679 km far from Addis Ababa and 117 km far from Bahir Dar. Ebinat is the administrative center of the district of Ebinat. Based on the information provided by the District Human Resources Administration Office, there are a total of 206,593 people, of which 102,996 are male and 103,297 are female. The structural plan of the district of Ebinat consists of 31 kebeles, 29 of which are rural and 2 of which are urban. It has 2 primary hospitals, 4 public health centers, 3 private medium-sized clinics, and 31 health centers.

### 2.2. Population

All women who gave birth in the past one year who have been living in the district were the source population, and those living in the selected rebels during the data collection period and who have been living in Ebinat for six months or more were the study population. Women who were less than 42 days postpartum, who did not start ANC follow-up at least once during pregnancy, and who were severely ill or unable to respond at the time of the interview (data collection) were excluded from the study.

### 2.3. Sample Size and Sampling Technique

The sample size was computed for both the first and second objectives, and the larger sample was taken as the sample size for this study. In detail, the sample size for the first objective was computed using a single population proportion formula as a 95% confidence interval, 5% accuracy, and 9.7% (magnitude of completion of the continuum of care) which was taken from the study conducted previously [16]. The final sample size for the first objective after applying a 10% nonresponse rate was 149, whereas the sample size for the second objective was calculated thru the double population proportion formula for the most significantly associated variables taken from the prevalent study conducted previously using the following information and assumptions: 95% confidence interval, 80% power, and 1 : 1 ratio of unexposed to exposed; considering pregnancy desire (planned) assumptions, the ultimate sample size became 504 (28), which was the largest sample size among calculated and taken as the sample size. The Ebinat district has a total of 31 kebeles, of which 8 kebeles were selected through a simple random sampling technique via the lottery method. Health registry books containing a list of women who have given birth in the last 12 months have been used as a sampling frame to identify the required sample size. The number of women delivered in each label was taken from the health extension workers' registration books. The calculated sample size has proportional allocation to each rebel, and a computer-generated simple random sampling technique was used to select the study units.

### 2.4. Operational Definition

*Maternity continuum of care*: main types of maternity health care services were that women receive ANC during pregnancy, institutional delivery assisted by SBA during childbirth, and PNC immediately after delivery until six weeks of delivery [6].

*Maternity continuum of care*: women who received the three components of maternity health care services that mean received ANC at least one time during pregnancy by a skilled provider, delivered at a health institution assisted by SBA (doctor, midwife, or nurse in the health institution) during childbirth, and received PNC services at least one time within 42 days during the postnatal period [6, 17–19]. Regarding PNC service utilization, all women who delivered at the health facility clearly obtained PNC service in the first 24 hours. However, it is difficult to conclude that they have awareness about the service of PNC, because purposively they went to the health facility for delivery service; fortunately, they would get the PNC services whether they need them or not. Thus, to say, a woman received PNC service at least if they return once for PNC service after being discharged to home. The women who received all these components were considered completed maternity continuum of care.

*Good knowledge*: women who are able to score ≥60% of total knowledge measuring items [20].

*Poor knowledge*: women who scored <60% of the total knowledge measuring items [20].

### 2.5. Data Collection Tools, Techniques, and Procedures

The pretested and structured questionnaire was used as a data collection tool that was prepared after the review of the relevant literature [1, 6, 10, 16, 21, 22]. The designed questionnaires were then changed from English to Amharic and returned to English to check the consistency of the questionnaire. Data were collected through face-to-face interviews. To ensure data quality, two days of training was provided to data collectors and supervisors on confidentiality of information, the rights of the respondent, informed consent, and interviewing techniques. Before proceeding to data collection, a pretest was performed on 5% of the total sample size of a randomly selected kebele in Adiss-Zemen District to ensure the validity of the tool and to standardize the questionnaire. The main investigators and supervisors checked and reviewed the questionnaires to check the completeness and consistency of the information collected daily.

Four data collectors and one supervisor were involved in the data collection process. The selected woman, who was not available at home, was revisited by the data collectors for the next two days, after having checked three times, and the women were not considered unavailable. The reliability of the questionnaire was checked using the Cronbach alpha value of 0.72. The data collection tool consists of six sections: sociodemographic characteristics, current marital profile, communication-related characteristics, and obstetrics-related factors, maternity care-related issues, and assessment of mother's knowledge towards completion of the maternity continuum of care.

### 2.6. Data Processing and Analysis

The data were visually checked for completeness and then encoded and entered into EpiData software (V. 3.1) and analyzed using SPSS software (V. 25). Descriptive analysis was performed, and the result was presented using text, tables, and graphs. A logistic regression model of the backward method has been used. The fitness of the model was tested using the Hosmer-Lemeshow fitness test of 0.39. Multicolinearity tests were carried out to see the correlation of independent variables with each other using variance inflation factors, and the value ranged from 1.04 to 1.55. A bivariable logistic regression was performed to see the presence or absence of a significant relationship of each independent variable with the dependent variable. During the bivariable analysis, variables with 95% CI at *P* value ≤ 0.25 were selected and entered into multivariate logistic regression for further analysis to determine those independently associated factors of the outcome variable through overcoming the effect of the confounding variables. The degree of association between independent and dependent variables was measured using an AOR with 95% CI. Finally, variables with a *P* value of <0.05 have been declared as statistically significant for the dependent variable.

## 3. Results

### 3.1. Sociodemographic Characteristics of the Study Respondents

The study was conducted among a total of 471 women who participated in the study, making the response rate 94%. The majority of 398 (84.5%) respondents were 20-34 years of age with the mean and standard deviation of 26.16 ± 4.97 years, respectively. All respondents were Amharan in ethnicity, and nearly three-fourths 366 (77.7%) of the respondents were from rural residences (Table 1).

### 3.2. Sociocultural and Husband-Related Factors

In this study, 287 (69.7%) partners knew about maternity health care services; of this, 286 (99.7%) knew about ANC services, around three-fourths of the respondents 328 (69.6%) were living a long distance from the health facility that takes >30 minutes traveled on foot to reach a health facility with a median of 50 and interquartile range of 45, and two-thirds of women's partners 269 (65.3%) were farmers.  Table 2 shows sociocultural and husband-related factors associated with continuum maternity care.

In this study, 411 (87.3%) women have used a modern type of contraceptive before the current pregnancy and two-thirds of the women 298 (63.3%) were multiparous (Table 3).

### 3.3. Maternity Health Care Service-Related Factors

**Figure 1:**
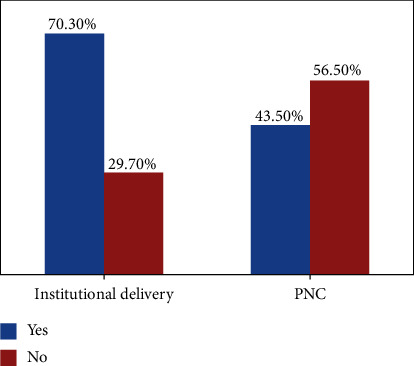
Maternity health care services utilization during pregnancy, delivery and PNC of their current child among women who gave birth in the last one year in northwest Ethiopia, 2020

All respondents used ANC service utilization. 70.30% of women have delivered in the institution, and 56.5% of women used PNC service (Figure 1).

**Figure 2:**
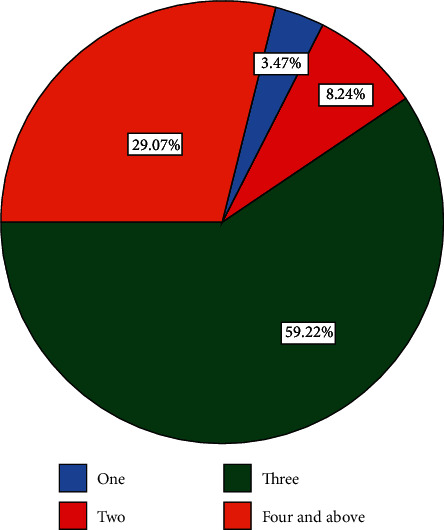
Number of ANC services utilization of the women in the last pregnancy of their current child among women who gave birth in the last one year in northwest Ethiopia, 2020

Regarding the number of ANC visits, all respondents who attended the ANC ward were receiving ANC services at the public health facility, either health centers or hospitals, but no one was receiving ANC services at the private health institution, and 273 (59.2%) of respondents had 3 times of ANC follow-up visit at the health facility by a skilled provider in their current child (Figure 2).

Concerning ANC counseling services, 436 (94.6%) respondents were counseled during ANC follow-up; of these, 410 (94%) were counseled for danger signs of pregnancy, 361 (82.8%) were counseled for the importance of nutrition during pregnancy, and 374 (85.8%) were counseled for birth preparedness and complication readiness strategy. About ANC services provided during pregnancy, all women who attended the ANC ward were measured blood pressure, taken blood and urine samples, and received iron and folic acid. Three-fourths 391 (84.8%) of the respondents have received tetanus toxoid vaccination, and 437 (94.8%) of the respondents have received HIV screening tests during ANC follow-up.

**Figure 3:**
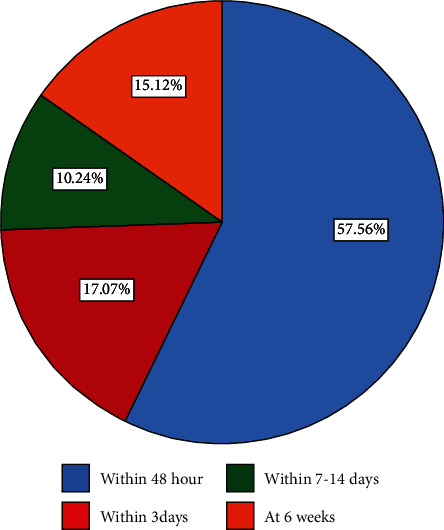
Time to receive PNC services in their current child among women who gave birth in the last one year in northwest Ethiopia, 2020

Regarding the place of postnatal checkup, all 205 (100%) of the respondents were receiving PNC services at the public health institution while 39 (19.0%) of the respondents were checking their PNC at home by health extension workers and 118 (57.6%) of the respondents received first postnatal checkup within 48 hours after delivery for their current child (Figure 3).

**Figure 4:**
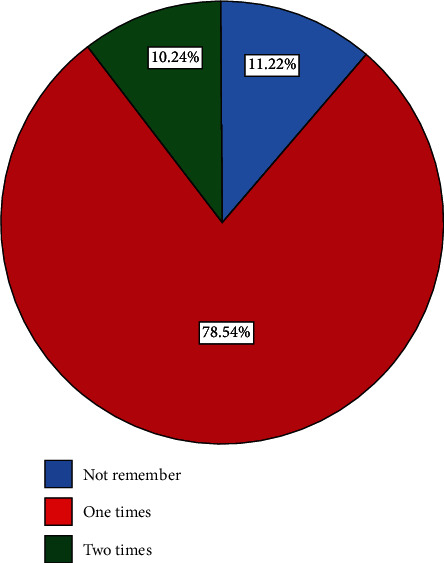
Number of PNC visits among women who gave birth in the last one year in northwest Ethiopia, 2020

As regards PNC counseling, among the respondents, 189 (92.2%) were counseled about postnatal care for their current child, three-fourths of them 135 (71.4%) were counseled about birth spacing and family planning services, 171 (90.5%) were counseled about how to breastfeed their baby, three-fourths of them 137 (72.5%) were counseled on hygiene, and 177 (93.7%) were counseled about child immunization schedule. Three-fourths of the respondents 161 (78.5%) had one-time PNC follow-up visit. The respondents were receiving PNC checkups a maximum of only two times; they failed to continue; WHO recommended a follow-up of at least four times during the postnatal period (Figure 4).

Two-thirds 323 (68.6%) of the respondents had good knowledge about the continuum of maternity health care services (Table 4).

**Figure 5:**
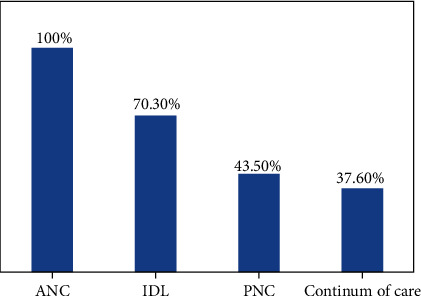
Continuum of care among women who gave birth in the last one year in northwest Ethiopia, 2020

Continuum of care women received ANC at least one time, institutionally delivered by an assisted skill birth attendant, and took PNC at least one time for their current child. The percentage of continuum care was 37.60 and ANC utilization was 100% (Figure 5).

Concerning the reason not to receive continuum of care, among women who did not deliver at a health facility, 126 (90.0%) of the respondents were due to distance, nearly three-fourths of them 98 (70.0%) were due to cultural reasons, and around two-thirds 91 (65.0%) of respondents were due to lack of support to take care of other children. For reasons of women who did not attend PNC services, 264 (99.2%) of respondents were due to lack of awareness about postnatal care services, 222 (83.5%) were due to distance, one-third of respondents 157 (59.0%) were due to did not think PNC services are necessary, and 63 (23.7%) of respondents were due to lack of support to take care of other children.

### 3.4. Factors Associated with Completion of Maternity Continuum of Care

Conferring to the bivariate logistic regression analysis, exposure variables including primary education, women completed secondary education and above, urban residence, used an ambulance as a means of transport, perceived time to reach a health facility < 30 minutes, autonomy of health care decision-making, and media exposure were significantly associated with the completion of the maternal continuum of care. In addition, 9 variables with *P* < 0.25 from the bivariate analysis were a candidate and recruited for multivariate analysis. Consequently, secondary and above educational status of women, urban residence, ambulance used as a means of transport, media exposure, perceived time to reach a health facility, and the overall knowledge of women were potential predictors of the completion of the maternity continuum of care. Conversely, the autonomy of health care decision-making and multipara women has been identified as protective factors.

In a multivariable analysis, the odds of women who completed secondary and higher levels of education were 2.75 times more likely to complete the maternity continuum of care (CMCC) compared to women who cannot read and write (AOR = 2.75, 95% CI 1.42-5.32); likewise, women who lived in urban residence were 2.45 times more likely to CMCC compared to women who lived in rural residence (AOR = 2.45, 95% CI 1.35-4.45); the odds of women who have used ambulance services to travel to a health facility for delivery services were 3.96 times more likely to CMCC than those who traveled on foot (AOR = 3.96, 95% CI 2.19-7.19).

Women who had been exposed to mass media at least once a week via either television or radio were 3.64 times more likely to complete maternity continuum care than women who did not use any type of media (**AOR** = 3.64, 95% CI 2.02-6.56); also, women who lived near a health facility that takes <30 minutes to reach were 3.22 times more likely to CMCC than those who lived far from a health facility that takes >30 minutes to reach (**AOR** = 3.22, 95% CI 1.84-5.63); likewise, women who had good knowledge on the continuum of care were 5.8 times more likely to CMCC (**AOR** = 5.81, 95% CI 2.89-11.70) than women who had poor knowledge (Table 5).

## 4. Discussion

The continuum of maternity health care services is critical for the integration of maternity and child health care services, and it is the primary strategy for reducing maternal and child mortality. In this study, women's education (secondary and higher), residence (urban), media exposure, mode of transportation (ambulance service), and perceived time to reach health facility were all examined. The facility (≥30 minutes) and overall knowledge of women about the continuum of care were important predictors of CCMC.

About 100% of women were receiving ANC during pregnancy; however, 62.4% of women were discontinued from the route of the continuum of care, comparable with the study done at [43]. The possible reason for this dropout might be an unpredictable onset of labor, cultural reasons, distance from the health facilities, and lack of awareness about PNC services; in this study, among the reasons of discontinuity of care 99.2% was not receiving PNC services due to the lack of awareness about postnatal care services. The magnitude of CCMC in this study was 37.6% (95% CI 33.1, 42.1), which is in line with the study done in Nepal 41% [19]. However, it is lower than the study conducted in northwest Ethiopia, 45% [18]. The discrepancies might be due to the cut point of the inclusion criteria of PNC. This study includes at least one returning to PNC service after discharge of institutional delivery, whereas the former study includes receiving PNC within 2 days that include the first 24 hours of PNC service of institutionally delivered women; even if it is a part of PNC service, the women are considered part of the delivery service since they came to the health institution for delivery care services [18].

Other studies in Cambodia, Egypt, and Nepal (50.4 percent, 45.7 percent, and 60 percent, respectively [3, 6, 21]) revealed a higher CCMC than ours. The inconsistencies could be attributed to PNC's eligibility criterium's cut point. This study includes at least one return of PNC service after discharge from institutional delivery, whereas previous studies included PNC services within 6 weeks, which includes the first 24 hours of PNC service for institutionally delivered women, and this could be attributable to a difference in sampling strategy, as all of those studies were survey studies, whereas this study employed a simple stratified sampling technique [3, 6, 21]; additionally, it might be associated with the variation in health care service accessibility and coverage in the study areas [18]. Because of technological advancements and methodological differences, the study in Egypt was an institutional-based study, which may have increased the magnitude of the continuum of care because participants had already visited a health facility for maternal health care services [21].

This finding is higher than the finding in southern Ethiopia 9.7% [16] and in northwest Ethiopia 12.1% [23]. The inconsistency might be due to time variation and the cut point of inclusion criteria of ANC in this study, including ANC service utilization at least once during pregnancy, while those studies include ANC service utilization at least 4 times during pregnancy [16, 23].

Our findings were greater than the study conducted in Ghana 8% and 10.3% [24, 25], in SSA 14% [17], in Cambodia 5% [1], and in south Asia 25% [17]. The discrepancy might be due to the cut point of inclusion criteria of ANC in this study, including ANC service utilization at least once during pregnancy, while those studies included ANC service utilization at least 4 times during pregnancy [1, 17, 24]; also, it might be due to the difference in the study area and time variation.

Women with secondary and above levels of educational attainment were 2.75 times more likely to complete a continuum of maternal health care service compared to those who were unable to read and write. This finding is supported by the finding in northwest Ethiopia [18], Egypt [21], Nepal [6], Pakistan [26], SSA, and south Asia [17]. This could be explained by the fact that educated women were more aware of the availability of maternity health care services, as education empowered women and increased their confidence and ability to choose to use maternal health care services [6, 27], since higher levels of educated women have a positive effect on exposure to media and autonomy of decision-making about their own health care services [18]. The positive relationship between education and completion of the continuum of care is that educated women are more knowledgeable about the use of the continuum of care pathway during pregnancy, delivery, and postnatal period and may have access to information to read health-related written materials and have better communication [8, 28].

Women who were living in an urban residence were 2.45 times more likely to CMCC compared to those who lived in rural residences. This finding is consistent with the finding in Ethiopia [8], in Nigeria [29], in Pakistan [26], in Nepal [6], and in SSA and south Asia [17]. The possible reason might be that women who live in urban residences have more chances to educate, have autonomy, and reach a health facility easily due to the availability of health institutions in the shorter distances [10]. There might be increased availability of infrastructures like the accessibility of transportation and better roads in urban residences [8]. There may be a difference in the availability of health care workers and health care providing materials between urban and rural. This indicated that urban residences are more advantaged in terms of the availability of health care providing materials and accessibility of health care professionals than rural residences [8]. Urban women may have more advantages to being exposed to mass media and less affected by cultural barriers related to maternity health care service [30]. Quality of maternity health care services and formal and informal education might be better in urban residences than rural residences [31–33].

Women who were exposed to media were 3.64 times more likely to CCMC compared to those who are not exposed to media. This finding is supported by the finding in northwest Ethiopia [18], in Holeta town, Ethiopia [27], in Nepal [6, 19], and in Pakistan [26]. This could be explained by the fact that respondents as well as the overall community media saturation were found to be a strong predictor of CCMC [34]. The possible reason might be respondents who had media exposure might have knowledge on the schedule and frequency of maternity health care services; media provides different educational messages and information about the importance of receiving maternity health care services [18]. The use of public media like watching television, listening to radio, and reading a newspaper at least once a week was more likely to CMCC [6]. The reason might be providing mass media campaign programs for respondents as well as for the general community was a key program to improve the coverage of a continuum of maternal health care services [35].

Women who used ambulance services to travel to a health facility for delivery services were 3.96 times more likely to CMCC compared to those who traveled on foot. This finding is supported by the finding in Ghana [24]. The possible reason might be the accessibility of better roads, transportation system, and availability of ambulance services making it easy to reach health facilities [8]. Accessibility of ambulance services for transportation of pregnant women for delivery care services might be saving time and reducing the tiredness of women than those traveling on foot. The women who lived in a rural residence cannot get access to regular public transport especially at night and during marketing days; due to this reason, traveling to the health facility for maternity health care services was difficult [24].

Women who lived near health facilities (taking <30 minutes) were 3.22 times more likely to CMCC compared to those who lived far from health facilities. This study is supported by the study in Ethiopia [27], Nigeria [29], Cambodia [1], and Lao PDR [36]. The possible reason might be women who live a closer distance from health facilities may not be challenged by the barriers of the second delay and easily reach the health facilities after the onset of labor. A study done in Tanzania revealed that 44.3% of women delivered at home due to the combination of distance to health institutions and lack of transportation to a traveled health facility for delivery services [37]. Long distance from home to the health facilities complicated by night labor with lack of transport to travel to health facilities for delivery services encouraged women to home delivery or discouraged them from going to health facilities [1].

Women who had good knowledge of the continuum of care were 5.8 times more likely to CMCC compared to those who had poor knowledge. This finding is supported by findings in rural Sierra Leone [38], in Cambodia [1], and in rural Bangladesh [39]. This could be explained by the fact that women who received health education about maternity health care services were part of an intervention package designed to promote maternity and child health [38]. The possible reason might be that women who had good knowledge of maternity health care services became familiar with the importance of receiving maternity health care services than their counterparts. Women who have good knowledge of each component of maternity health care services might receive the service based on its schedule and frequency than those who have poor knowledge.

## 5. Conclusion

The magnitude of the completed pathway of the continuum of maternity health care services in the study area has been found to be less practiced and was shallow; this indicated that the women were not receiving the maximum possible health benefit from existing maternal health care service packages. The highest discontinuation rate was observed during the PNC period (58.8%). Distance, lack of transportation, and dearth of awareness about PNC services contribute to the low coverage of PNC; all these were the main reasons for the discontinuation of the pathway of the continuum of care. Women's education level, urban residence, media exposure, ambulance transportation services, perceived time to reach health facilities, and overall knowledge of women about the continuum of care were important predictors of CMCC, and attention is being paid to these predictors, and comprehensive awareness-raising, promotion activities, and health education of women shall be implemented at the community and health facility levels.

## Figures and Tables

**Table 1:** Sociodemographic characteristics of women who gave birth in the last one year in northwest Ethiopia, 2020 (*n* = 471).

Variables	Category	*N* (%)
Age in years	<20	34 (7.2)
	20-34	398 (84.5)
	≥35	39 (8.3)
Occupation	Housewife	392 (83.2)
	Employed	34 (7.2)
	Merchant	32 (6.8)
	Others^∗^	13 (2.8)
Level of education	Unable to read	220 (46.7)
	Can read and write	35 (7.4)
	Primary education	98 (20.8)
	Secondary and above	118 (25.1)
Religion	Orthodox	429 (91.1)
	Muslim	27 (5.7)
	Others^∗∗^	15 (3.2)
Marital status	Single	34 (7.2)
	Married	411 (87.3)
	Divorced	19 (4.0)
	Widowed	7 (1.5)

^∗^Other = 9 were students and 4 were daily laborers. ^∗∗^Other = 12 protestant and 3 catholic.

**Table 2:** Sociocultural and husband-related factors of women who gave birth in northwest Ethiopia, 2020 (*n* = 471).

Variables	Category	*N* (%)
Occupation of partner	Merchant	54 (13.1)
	Farmer	269 (65.3)
	Employed	89 (21.6)
Partner education level	Unable to read	114 (27.7)
	Can read and write	100 (24.3)
	Primary education	84 (20.4)
	Secondary education	60 (14.6)
	College and above	54 (13)
Means of transportation	On foot	271 (57.5)
	Public transport	83 (17.6)
	Ambulance	117 (24.8)
Autonomy of women	Yes	344 (73.0)
	No	127 (27.0)
Media exposure of women	Yes	152 (32.3)
	No	319 (67.7)
Types of media	Television	111 (23.6)
	Radio	41 (8.7)
Partners knew about delivery services	Yes	283 (97.9)
	No	6 (2.1)
Partners knew about PNC services	Yes	218 (75.2)
	No	72 (24.8)

**Table 3:** Obstetrics-related factors of women who gave birth in the last one year in northwest Ethiopia, 2020 (*n* = 471).

Variables	Category	*N* (%)
Number of total parity	Prim-para	114 (24.2)
	Multiparous	298 (63.3)
	Grand multiparous	59 (12.5)
Number of children	One	115 (24.4)
	Two	132 (28.0)
	Three	96 (20.4)
	Four	69 (14.6)
	≥Five	59 (12.5)
Pregnancy neediness	Yes	457 (97.0)
	No	14 (3.0)

**Table 4:** Knowledge of women about maternity health care services among women who gave birth in the last one year in northwest Ethiopia, 2020.

Variables	Category	*N* (%)
ANC knowledge	Good knowledge	448 (95.1)
	Poor knowledge	23 (4.9)
Delivery knowledge	Good knowledge	346 (73.5)
	Poor knowledge	125 (26.5)
PNC knowledge	Good knowledge	184 (39.1)
	Poor knowledge	287 (60.9)

**Table 5:** Factors affecting completion of maternity continuum of care among mothers who gave birth in the last one year in northwest Ethiopia, 2020.

Variables		Completion of continuum of maternity care		COR (95% CI)	AOR (95% CI)
		Yes (%)	No (%)		
Educational status	Cannot read	45 (20.5)	175(79.5)	1	1
	Can read and write	17 (48.6)	18 (51.4)	3.67 (1.75-7.69)	2.31 (0.82-6.02)
	Primary education	37 (37.8)	61 (62.2)	2.36 (1.40-3.98)	2.17 (1.11-4.23)^↑^
	Secondary and above	78 (66.1)	40 (33.9)	7.58 (4.59-12.54)	2.75 (1.42-5.32)^↑^
Residence	Urban	76 (72.4)	29 (27.6)	6.88 (4.23-11.17)	2.45 (1.35-4.45)^↑^
	Rural	101 (27.6)	265 (72.4)	1	1
Means of transportation	On foot	73 (26.9)	198 (73.1)	1	1
	Public transport	29 (34.9)	54 (65.1)	1.46 (0.86-2.46)	1.10 (0.54-2.24)
	Ambulance	75 (64.1)	42 (35.9)	4.84 (3.05-7.70)	3.96 (2.19-7.19)^↑^
Time to reach HF	<30 minutes	90 (62.9)	53 (37.1)	4.70 (3.10-7.15)	3.22 (1.84-5.63)^↑^
	≥30 minutes	87 (26.5)	241 (73.5)	1	1
Autonomy	Yes	153 (44.5)	191 (55.5)	3.44 (2.10-5.63)	0.89 (0.46-1.71)
	No	24 (18.9)	103 (81.1)	1	1
Media exposure	Yes	108 (71.1)	44 (28.9)	8.89 (5.73-13.81)	3.64 (2.02-6.56)^↑^
	No	69 (21.6)	250 (78.4)	1	1
Prepregnancy contraindication	Yes	161 (39.2)	250 (60.8)	1.77 (0.98-3.25)	1.51 (0.66-3.49)
	No	16 (26.7)	44 (73.3)	1	1
Number of parities	Prim-para	54 (47.4)	60 (52.6)	1	1
	Multipara	100 (33.6)	198 (66.4)	0.56 (0.36-0.87)	0.70 (0.38-1.26)
	Grand multipara	23 (39.0)	36 (61.0)	0.71 (0.38-1.35)	1.90 (0.79-4.60)
Overall knowledge	Good	159 (49.2)	164 (50.8)	7.99 (4.68-13.64)	5.81 (2.89-11.70)^↑^
	Poor	18 (12.2)	130 (87.8)	1	1

COR = crude odds ratio; AOR = adjusted odds ratio. ^↑^Statistically significant at *P* < 0.05.

## Data Availability

The data used to support the findings of this study are included in the article.

## Abbreviations

ANC: Antenatal care
AOR: Adjusted odds ratio
BPCR: Birth preparedness and complication readiness
CI: Confidence interval
COR: Crude odds ratio
CCC: Complete maternity continuum of care
EDHS: Ethiopian Demographic and Health Survey
FP: Family planning
HEW: Health extension worker
MMR: Maternal mortality ratio
NGO: Nongovernmental organization
OR: Odds ratio
PNC: Postnatal care
SBA: Skill birth attendant
SPSS: Statistical package for the social sciences
TT: Tetanus toxoid.
